# Suspicion de purpura rhumatoïde post vaccination antigrippale A (H1N1) compliqué d’invagination intestinale aiguë chez un enfant de quatre ans

**Published:** 2010-12-09

**Authors:** Lamiae Chater, Khalid Khattala, Hanane Guerrouj, Ibtissam Bouamama, Mohamed Rami, Moulay Abderrahmane Afifi, Youssef Bouabdallah

**Affiliations:** 1 Service de chirurgie pédiatrique, CHU Hassan II Fès, Maroc

**Keywords:** Purpura rhumatoide, vaccin antigrippal A, H1N1, invagination, enfant

## Abstract

**Abstract:**

Le purpura rhumatoïde représente la vascularite immunoallergique la plus fréquente de l’enfant. Sa survenue dans les suites d'une vaccination antigrippale est exceptionnelle. Nous rapportons l’observation d’une fille de 4ans qui présente une suspicion de purpura rhumatoïde post vaccination antigrippale A(H1N1) compliqué d’invagination intestinale aiguë ayant bénéficié d’une réduction chirurgicale. L’évolution était favorable avec un recul de un an. L’invagination intestinale aiguë constitue la complication digestive la plus redoutable du purpura rhumatoïde. Dont le diagnostic doit être toujours évoqué devant des douleurs abdominales aigues chez un enfant porteur de cette vascularite.

## Introduction

Les vascularites post-vaccinales sont rares, surtout chez l’enfant, et dont le mécanisme physiopathologique est peu connu. Le purpura rhumatoïde constitue la vascularite la plus fréquente pouvant se compliquer d’invagination intestinale aiguë.

## Patient et observation

Il s’agit de l’enfant O. B âgée de 4 ans. Admise aux urgences dans un tableau de douleurs abdominales intenses d’installation brutale accompagnées de vomissements bilieux et de rectorragies apparues quelques heures avant son hospitalisation évoluant dans un contexte d’apyrexie. La maman rapporte également la notion d’arthralgies avec une éruption purpurique intéressant les cuisses et les jambes quatre jours avant son admission. On note également dans ses antécédents la notion de vaccination antigrippale A(H1N1) dix jours avant l’apparition de sa symptomatologie. La radiographie d’abdomen sans préparation a montré la présence de niveaux hydro-aériques de type grêliques, et l’échographie abdominale a objectivé une image évoquant un boudin d’invagination.

**Figure 1:**
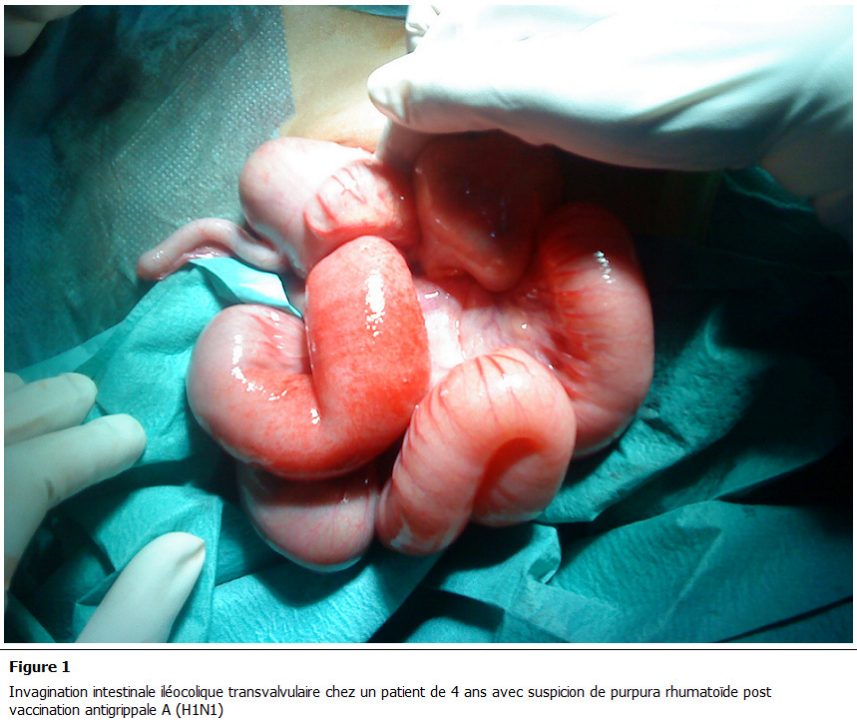
invagination intestinale iléocolique transvalvulaire chez un patient de 4 ans avec suspicion de purpura rhumatoïde post vaccination antigrippale A (H1N1)

**Figure 3:**
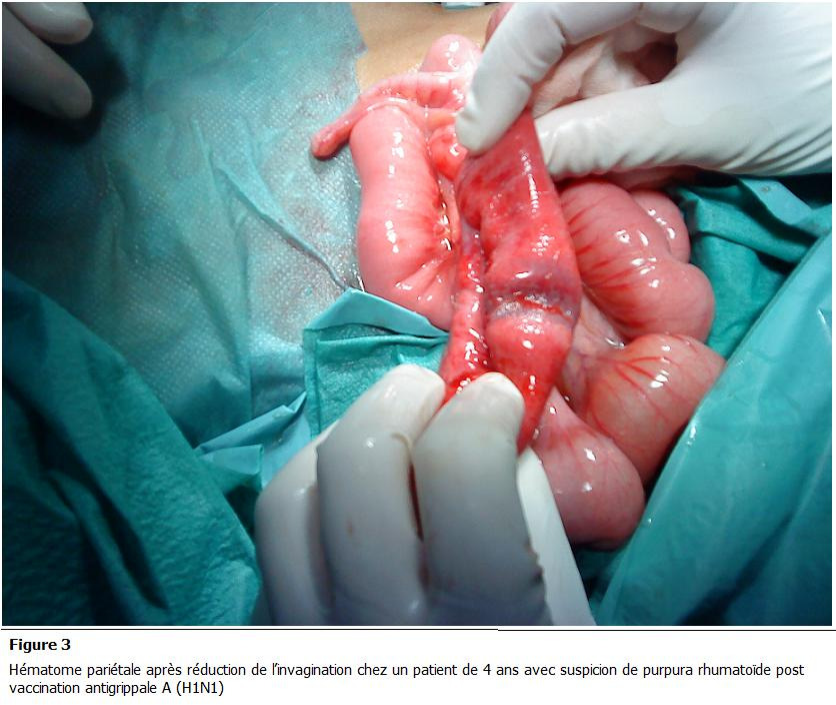
hématome pariétale après réduction de l’invagination chez un patient de 4 ans avec suspicion de purpura rhumatoïde post vaccination antigrippale A (H1N1)

La laparotomie médiane a été réalisée ayant permis de découvrir une invagination iléo-colique transvalvulaire avec un aspect oedématié et ecchymotique de l’intestin qui est le siège d’un hématome pariétal (figure 1 et 2), une désinvagination manuelle difficile a été réalisée (Figure 3) avec une bonne évolution clinique.

## Discussion

Les mécanismes physiopathologiques exacts des vascularites associées aux vaccins sont peu clairs. La première hypothèse “immunologique” de type complexes immuns circulants suppose le rôle d’un antigène vaccinal agissant comme starter. Ce mécanisme paraît proche de la pathogénie des périartérites noueuses liées au virus de l'hépatite B (VHB) [1]. Cependant, dans la majorité des vascularites, l’agent étiologique n’est pas connu. La vaccination étant, d’une certaine manière, supposée mimer l’infection, l’hypothèse de sa responsabilité dans certaines maladies immunologiques humaines a été récemment remise en première ligne. Patel et al. relèvent un fort taux d’anticorps dirigés contre l’antigène vaccinal utilisé. Guillevin et Levy [2] ont observé un test de dégranulation de basophiles humains positifs au cours d’une réaction d’hypersensibilité après une vaccination antigrippale. Dans la littérature, les deux vaccins le plus souvent incriminés dans le déclenchement d’une vascularite sont les vaccins anti-VHB et antigrippaux, mais ce sont également les vaccins les plus prescrits chez l’adulte. Vinceneux et al. [3] reprennent 14 observations de vascularites, avec ou sans preuve histologique, après une vaccination antigrippale [4,5]. Il s’agit de sept femmes et de sept hommes, d’âge moyen 62 ans, dont les symptômes sont apparus en moyenne neuf jours après une vaccination. Dans notre cas il s’agit d’une fille de 4 ans, dont les symptômes sont apparus 10 jours après la vaccination.

Le purpura rhumatoïde représente la vascularite immuno-allergique la plus fréquente de l'enfant (maximum entre 4 et 7 ans) avec une incidence de 20/100000, mais vraisemblablement infectieuse (caractère épidémique et saisonnier fréquent) dont la sémiologie associe dans un contexte fébrile des signes cutanés, articulaires, digestifs et un risque rénal avec un état général souvent conservé.

Dans 25 à 90% des cas, on retrouve un facteur déclenchant; le plus souvent une infection bactérienne (streptocoque, *Mycoplasma* pneumoniaie, staphylocoque, clostridium...) ou virale (Parvovirus B19, EBV, adénovirus, virus ourlien, HIV), plus rarement, une infection parasitaire, piqure d'insecte, voire toxique (cocaïne), parfois une prise médicamenteuse (antibiotiques, anti inflammatoires non-stéroïdiens (AINS), Inhibiteurs de l’enzyme de conversion (IEC)) et exceptionnellement une vaccination.

L’invagination intestinale aiguë est la complication chirurgicale la plus fréquente du purpura rhumatoïde. Sa physiopathologie est en rapport avec les perturbations péristaltiques secondaire à l’œdème et aux hématomes pariétaux.

Le diagnostic de ces invaginations est souvent difficile d’où l’intérêt de l’échographie. Le traitement de ces complications est souvent chirurgical. L’évolution est en général favorable.

## Conclusion

Bien que l’existence de vascularites postvaccinales reste toujours à démontrer formellement, nous ajoutons à la liste déjà existante ce cas de purpura rhumatoïde postvaccination antigrippale A (H1N1) chez l’enfant. Le faible nombre de cas publiés de vascularites post-vaccinales (n’excédant pas la centaine chez l’adulte et très exceptionnel chez l’enfant), ne remet pas en cause l’innocuité des vaccins, mais la connaissance de telles complications mérite d’être connue afin d’une part, d’éviter de nouvelles immunisations pouvant avoir des conséquences plus sévères et d’autre part d’éviter d’aggraver ou de réactiver une vascularite préexistante.

L’invagination intestinale aiguë constitue la complication chirurgicale la plus fréquente du purpura rhumatoïde qui doit être cherché avec prudence pour éviter la perforation.

## Competing interests

The authors declare no competing interests.

## Remerciements

Je remercie tous les auteurs qui ont participé à l’élaboration de ce travail

## Conflits d’intérêts

Les auteurs déclarent n’avoir aucuns conflits d’intérêts.

## Contributions des auteurs

Lamiae Chater, Khalid Khattala et Hanane Bouamama ont participé à la prise en charge du patient, la prise des photos et la rédaction de l’article, Aziz Elmadi et Mohamed Rami ont participé à la recherche bibliographique, et Moulay Abderrahmane Afifi et Youssef Bouabdallah ont participé à la prise en charge de l’enfant.

## Figures and Tables

**Figure 2:**
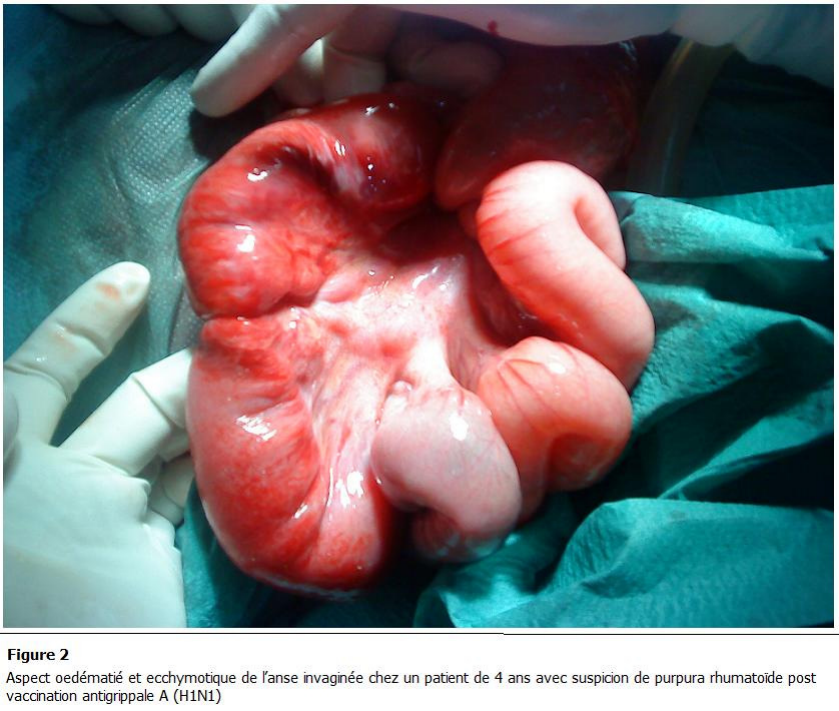
aspect oedématié et ecchymotique de l’anse invaginée chez un patient de 4 ans avec suspicion de purpura rhumatoïde post vaccination antigrippale A (H1N1)
